# Infants’ gaze exhibits a fractal structure that varies by age and stimulus salience

**DOI:** 10.1038/s41598-020-73187-w

**Published:** 2020-10-14

**Authors:** Isabella C. Stallworthy, Robin Sifre, Daniel Berry, Carolyn Lasch, Tim J. Smith, Jed T. Elison

**Affiliations:** 1 Institute of Child Development, University of Minnesota, Minneapolis, USA; 2 Department of Psychological Sciences, Birkbeck University of London, London, UK; 3 Department of Pediatrics, University of Minnesota, Minneapolis, USA

**Keywords:** Psychology, Human behaviour

## Abstract

The development of selective visual attention is critical for effectively engaging with an ever-changing world. Its optimal deployment depends upon interactions between neural, motor, and sensory systems across multiple timescales and neurocognitive loci. Previous work illustrates the spatio-temporal dynamics of these processes in adults, but less is known about this emergent phenomenon early in life. Using data (n = 190; 421 visits) collected between 3 and 35 months of age, we examined the spatio-temporal complexity of young children’s gaze patterns as they viewed stimuli varying in semantic salience. Specifically, we used detrended fluctuation analysis (DFA) to quantify the extent to which infants’ gaze patterns exhibited scale invariant patterns of nested variability, an organizational feature thought to reflect self-organized and optimally flexible system dynamics that are not overly rigid or random. Results indicated that gaze patterns of even the youngest infants exhibited fractal organization that increased with age. Further, fractal organization was greater when children (a) viewed social stimuli compared to stimuli with degraded social information and (b) when they spontaneously gazed at faces. These findings suggest that selective attention is well-organized in infancy, particularly toward social information, and indicate noteworthy growth in these processes across the first years of life.

## Introduction

### Visual attention

We deploy gaze in a dynamic fashion, fixating objects and people for sometimes many seconds at a time while also exhibiting oculomotor activity on the order of milliseconds. Human visual attention reflects the complex integration of information across an array of systems^1^. From a dynamic systems perspective, this real-time integration is thought to reflect the ‘soft assembly’ of neural, motor, and visceral processes, where the collective functioning of the system is an emergent (i.e., self-organized) property of its highly interactive constituent parts^2–4^. Specifically, by ‘highly interactive’ we mean that the role of each constituent component can only be understood in the context of its functioning with the other components of the system^3^.

The visual attention system develops rapidly on multiple timescales during infancy and toddlerhood and involves both bottom-up (i.e., stimulus-driven) and top-down (i.e., internally driven) influences. Infants are born with a rudimentary capacity for alertness, which is driven mainly by exogenous events and supported by primarily subcortical neural pathways and increases significantly within the first couple months of life^5^. Spatial orienting and attention disengagement abilities establish by around 6 months of age, along with the emergence of attention to features of objects and anticipatory eye movements^6^. Finally, in the latter half of the first year of life extending through about age 3 years, more endogenous volitional attention develops, including attentional inhibition abilities and more focused looking^5^.

Infants do not attend to all aspects of their surroundings equally—past work has elucidated adaptive predispositions to visually engage with social features in the environment such as faces^7^ face-like stimuli^8^, and biological motion^9^. Faces are especially salient beginning moments after birth and attention to faces becomes more refined with experience and the development of cortical influence^10^. Social features continue to capture attention within the first year of life^11^ and throughout the preschool years in increasingly sophisticated ways^12^.

In short, the burgeoning visual attention system is dynamic, multidimensional, and sensitive to context. It encompasses many interacting sub-components with different organizational structures as well as many functional and developmental timescales^13,14^. As such, the development of this system must be considered with respect to its interaction-dominant, non-linear organization^15^. Researchers have long recognized the importance of examining the temporal processes of visual attention development, as it can reveal new information about the relationship between global average looking times and the processes that give rise to them (e.g.,^16^). Despite this qualitative observation, little work has quantified the dynamic organization of infants’ gaze behavior (with the exception of^17^). The current study fills this gap using a dynamic mathematical approach that is particularly well-suited for quantifying interaction-dominant emergent phenomena^18^.

### Fractal organization

Given the complexity of the developing visual system, we utilized time-series *fractality*, an increasingly common approach for characterizing the multiscale organization of complex, psychobiological processes^19^. Fractal structures are characterized by nested patterns of variability that are scale-invariant, i.e., apparent across multiple measurement scales^20^, resulting in a *self-similarity* over time. These structures result from simple local rules that interact to produce highly complex, dynamic organizational structures^21^. Fractal organization is ubiquitous in nature (e.g., in mountain ranges and riverbeds^22^) and human physiology (e.g., cardiac anatomy^23^), and is thought to be a critical feature of healthy cognitive and psychobiological systems^24–26^.

Mathematically, fractal structures reflect a proportional relationship between the power (i.e., size of change) and the frequency (i.e., how frequently changes of that size occur) of variation within a time-series^20^. This relation suggests self-similarity and scale-invariance, such that the variability observed for the system’s behavior will be proportionally identical, irrespective of the length of the overall scale. In other words, the pattern of variability should look the same as one magnifies the resolution in and out of increasingly micro- or macroscopic temporal scales.

**Figure 1:**
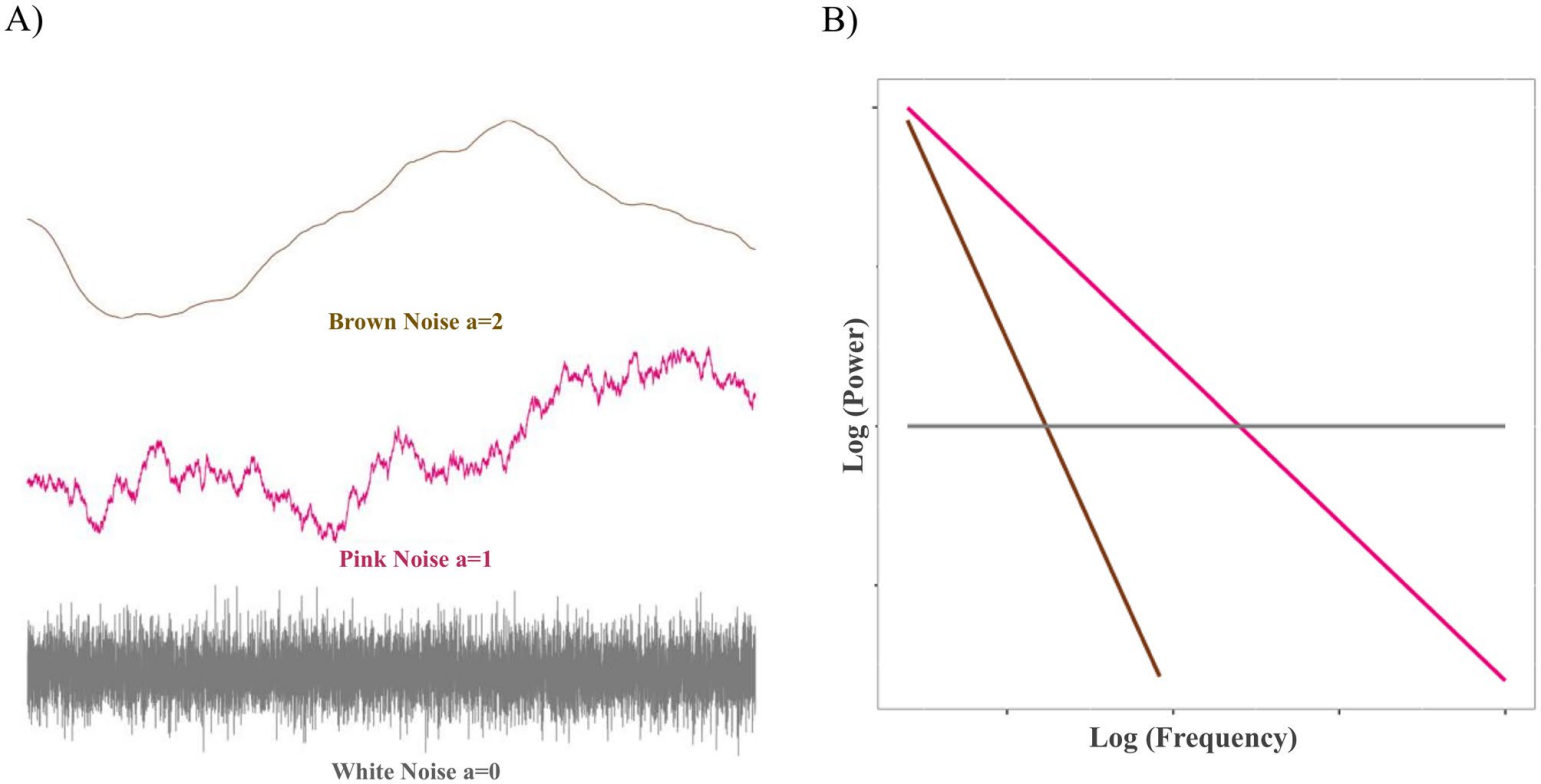
(**A**) Sample time-series and (**B**) the relationship between power and frequency for pink noise shown in pink; white noise shown in grey; and brown noise shown in brown.

The extent to which a time-series is characterized by this proportional ideal is commonly discussed with respect to the *color* of the time-series organization (Fig. 1A). Here, the theoretically optimally proportional relation between power and frequency of time-series variation is known as *pink* noise^27^. In the frequency domain, this corresponds to a negative relation of 1, on a log–log scale (Fig. 1B). Pink noise gets its name from the fact that its variability falls in the middle of a continuum of noises—white to red—in which scale units correspond to mathematical organization of the series to reflect the underlying organizational structure of the system. The ‘power law’ relation of pink noise that approaches perfect proportionality implies self-similarity, scale invariance, and autocorrelation functions indicating long-term memories^28^.

Given these properties, pink noise organization is considered to be optimal as it is thought to reflect system dynamics that are highly self-organized, yet also flexible to change given that its structure is not overly rigid or random. ‘Self-organized’ refers to the property through which dynamic systems exhibit organizational structures that are not driven by any one subcomponent or lower level of cause and effect^34,26^. States of a system may be shaped by control parameters but cannot be reduced to them. In biological systems, self-organization may coordinate the many bodily processes across timescales^26^. Self-organization is thought to require interaction-dominant dynamics, through which local interactions heavily influence each other (as opposed to more individual more component-dominant dynamics), thereby producing statistically self-similar patterns of variability, such as those as indexed by fractal metrics.

At the low end of the noise distribution, *white-noise* time-series (or fractional Gaussian noise^27^) reflect completely random variation; changes of all sizes occur at the same frequency and share no relation to the other observations of the series^20^. Given the lack of self-similarity, scale-invariance, or memory in the system, white noise is thought to reflect systems lacking internal organization.

At the opposite end of the distribution, red noise—more commonly called *brown noise* or fractional Brownian motion*—*is characterized by time-series in which each observation represents a random change from the prior observation. Red/brown noise represents the integration of a white-noise series, such that it comprises the cumulative sum of the random white-noise shocks. As such, each observation contributes information to the entirety of the subsequent observations, leading to an ‘infinite memory’ in the system, in which autocorrelation remains the same, irrespective of the temporal lag between observations. This infinite memory is thought to reflect the behavior of systems that are highly constrained and rigid to change.

### Fractal dynamics of cognitive processes

Thus, collectively, fractal structure is thought to reflect the coordination of multiple interacting systems organized in an optimally flexible way (pink noise), without being too rigid (brown noise) or too random (white noise). Empirically, pink noise organization has been found for an array of cognitive tasks and modalities in adults—response times during shape discrimination^24^, mental rotation and translation^29^, bi-stable perception^18^, word naming^26^, and simple stimulus detection^30^; tracing patterns during forced-trace problem-solving^31^ and circle-drawing^32^, as well as eye movements during free viewing^33^, challenging visual search^34^, and text reading^35^.

Conversely, white noise variation has been found for time-series of gaze patterns that have been randomly shuffled (i.e., preserving the time-series values but disrupting their temporal relations^20^) and for the non-biological simulated “fixations” produced by a fake eye^20^. Further, relatively whiter noise has been found to be a marker of systems operating under more unpredictable constraints, for example when instructed to avoid previous cognitive biases^36^, while straining working memory^37^, or engaging in multi-tasking^38^. Both white and brown noise are also characteristic of aging and pathophysiological systems^39,40^.

### The current study

The current study employed fractal metrics to quantify the self-organization of infants’ gaze patterns over time and space throughout the first 3 years of life. Given the importance of context in shaping dynamic attention systems, participants (N = 190, age 3–35 months) watched movies of varying degrees of salience while an eye-tracker recorded their eye-movements: movies of women dancing to lively music while waving toys, versions of these videos in which the social information was pixelated, and brief presentations of dynamic audio-visual attention cues to serve as a baseline.

We used Detrended Fluctuation Analysis (DFA^19,41^; see “Methods”) that produces a scaling exponent, α, representing the proportional relation between patterns of variation within a time-series across a wide array of potential timescales (i.e., time segments of different lengths). Given the proportional properties of log–log relations, pink noise is indicated to the extent to which this relation approaches 1 on a log–log scale. That is, patterns of variability are proportionally identical, irrespective of timescale. Pink noise sits on a continuum between white noise (α ~ 0.5) and brown noise (α ~ 1.5), with white noise characterizing systems lacking organization, and brown noise characterizing overly rigid and deterministic systems.

We calculated α values from time-series created from infants’ raw eye-gaze data over time and space (see “Methods”) as they viewed the different movies. We then examined age-related change in overall α values, as well as changes in α values as a function of stimulus content across these three conditions (Social, Pixelated, Attention Cue).

### Hypotheses regarding the fractal organization of gaze

We propose three hypotheses regarding the development of gaze complexity within the first 3 years of life. First, given past work suggesting that healthy physiological systems exhibit organizational structure within the pink noise range^42^, as well as evidence that adults’ gaze patterns have an underlying pink noise^15,20,34,43^, we hypothesized that infants’ gaze patterns would, on average, be within the optimally flexible fractal range (pink noise, *α* ~ 1).

Second, we hypothesized that infants’ gaze patterns would become increasingly complex over the first 3 years of life (as indexed by an increase in *α* within the pink noise range), reflecting known developmental advances in the visual attention system (e.g., increased oculomotor control, ability to suppress orienting to salient peripheral stimuli, production of anti-saccades, and recruitment of top-down executive control functions^5,13,14^). We reasoned that these advances require increased coordination between systems, which should be reflected by a fractal organization of gaze patterns increasingly closer to pink noise values (i.e., *α* ~ 1).

Our third hypothesis concerns change in the fractal structure of gaze as a function of context. Given the ubiquity of pink noise, the interpretability of findings relies on specific predictions of how α should change as a function of task constraints^26^. Past work with adults has suggested that fractal structure might reflect aspects of vigilance and task engagement^24–26^. While measuring engagement in infants is challenging, past work has demonstrated that infants’ have strong attentional preferences to faces beginning moments after birth^44^, and that this preference persists through the first years of life^45,11^. Thus, we hypothesized that the infant visual system may be tuned to be especially organized and flexibly responsive—evidenced by relatively more self-organized fractal structure—while viewing movies in the Social condition *and* while attending to faces within the movies (e.g., stimuli known to be engaging). We employed linear mixed effects models to test the above hypotheses, with the *α* values for each gaze-based time-series as our primary dependent variable.

## Results

### Fractal organization of the infant visual system

Scaling exponent *α* values were approximately normally distributed (skew = 0.11, kurtosis = 4.22), with a mean of 0.84 range 0.12–1.34. The kurtosis value is within the range of a normal distribution for large sample sizes^46^. Ninety-one percent of the time-series across all 3 conditions fell within what is thought to be the optimally flexible fractal, pink noise range (*α* ~ 0.7 to 1.0^19,20^). The average R^2^ values for the linear model fit to the relations between log(frequency) and log(power) was 0.985 (interquartile range 0.01), suggesting that a linear power-law provided good model fit.

### Age-related change and effects of stimulus type on gaze complexity

Our baseline linear mixed effects model found that *α* values increased significantly with age (Δ-2LL = 21.1, Δdf = 1, p < 0.001) by about 0.0022 units a year (remaining within the pink noise range). Age alone predicted about 2.3% of the total variance in *α* values. Even the youngest infants in our sample (3-month-olds) demonstrated gaze patterns in the range typically considered to imply fractality (*α* = 0.79).

After adding a series of quality-control covariates to account for the quality of the data and eye-tracking recording (as detailed in “Methods”), we assessed the effects of stimulus condition on *α* values. Our best-fitting model indicated that, on average and irrespective of age, *α* values were 0.023 units lower (10% of a SD, i.e., less organized) while infants were watching Pixelated movies (Δ-2LL = 4.6, Δdf = 1, p = 0.0024), and 0.030 units lower while infants watched the audio-visual attention cues (Δ-2LL = 7.8, Δdf = 1, p < 0.001), compared to when they were watching the Social movies. There were no significant differences in *α* values between the Pixelated and audio-visual attention cues (*p* = 0.21).

**Figure 2:**
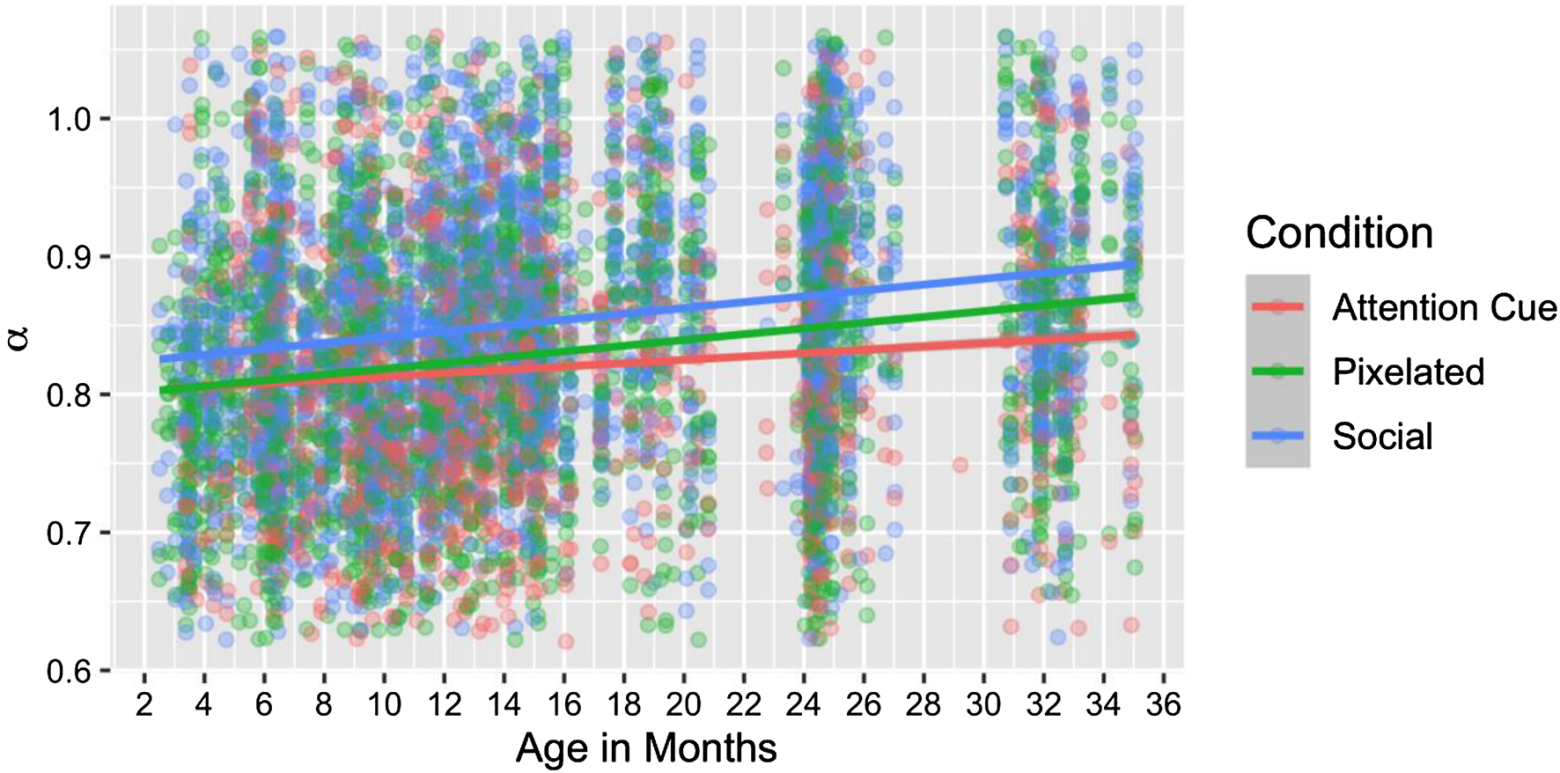
Age-related increase in *α* values (reflecting increased fractal organization of gaze patterns) by the type of stimulus being viewed.

A significant Condition x Age interaction (Δ-2LL = 6.7, Δdf = 1, p < 0.001; as shown in Fig. 2) indicated that growth in the fractal organization of infants’ eye gaze differed across stimulus type. Tests of the simple slopes suggested that positive growth was limited to the Social and Pixelated conditions (i.e., Social *b* = 0.0013, *p* = 0.0024; Pixelated *b* = 0.0010, *p* = 0.018; Attention Cue *b* = 0.00014, *p* = 0.78). We found a significantly greater rate of growth in *α* for the Social condition compared to the Attention Cue condition (Δ-2LL = 6.7, Δdf = 1, p < 0.001 while differences in the growth trajectories of *α* between the Pixelated and Attention Cue condition were non-significant. The rate of growth in *α* was comparable between Pixelated and Social conditions. The final model, Model 7 shown in Table 1, accounted for 18.36% of the total variance in *α* values.

**Table 1:** Linear mixed effects model table of model evidence displaying the effects of age and each of the stimulus conditions on *a* values with the Social condition as the reference event.

	Model 1	Model 2	Model 3	Model 4	Model 5	Model 6	Model 7	Model 8
**Fixed effects**								
Intercept	0.824*** (0.005)	0.824*** (0.005)	0.824*** (0.005)	0.830*** (0.005)	0.851*** (0.005)	0.851*** (0.005)	0.851*** (0.005)	0.824*** (0.004)
Person-level								
Avg Prec				− 0.198*** (0.014)	− 0.198*** (0.014)	− 0.201*** (0.014)	− 0.200*** (0.014)	− 0.201*** (0.014)
Avg age				− 0.001* (0.001)	− 0.001* (0.001)	− 0.001* (0.001)	− 0.001* (0.001)	− 0.001* (0.001)
Visit-level								
Age		0.002*** (0.000)	0.002*** (0.000)	0.001** (0.000)	0.001** (0.000)	0.001** (0.000)	0.001** (0.000)	0.001 (0.000)
ET Prec				− 0.146*** (0.018)	− 0.145*** (0.018)	− 0.145*** (0.018)	− 0.146*** (0.018)	− 0.146*** (0.018)
Movie-level								
Pixelated					− 0.023*** (0.006)	− 0.023*** (0.006)	− 0.023*** (0.006)	− 0.011*** (0.003)
Atten cue					− 0.030*** (0.006)	− 0.030*** (0.006)	− 0.030*** (0.006)	− 0.015*** (0.003)
Age: Pix						0.000 (0.000)		0.000 (0.000)
Age:AtCue							− 0.001*** (0.000)	− 0.001*** (0.000)
**Random effects**								
Btw-person								
Intercept	0.001	0.001	0.001	0.000	0.000	0.000	0.000	0.000
Age			0.000	0.000	0.000	0.000	0.000	0.000
Btw-VISIT								
Intercept	0.002	0.002	0.002	0.001	0.001	0.001	0.001	0.001
Btw-movie								
Intercept	0.000	0.000	0.000	0.000	0.000	0.000	0.000	0.000
Residual	0.008	0.008	0.008	0.008	0.008	0.008	0.008	0.008
**Model fit indices**								
AIC	− 16,889.6	− 16,929.7	− 16,934.6	− 17,114.8	− 17,126.5	–17,124.7	− 17,138.4	− 17,137.6
BIC	− 16,854.1	− 16,887.1	− 16,877.8	− 17,036.8	− 17,034.3	− 17,025.4	− 17,039.0	− 17,031.1
Log likelihood	8449.8	8470.9	8475.3	8568.4	8576.3	8576.4	8583.2	8583.8

Model 7 is the final, best-fitting model. Standard errors displayed in parentheses and significant effects are bolded for emphasis.

### Visual complexity and gaze to faces

Multi-level logistic regression was used to examine whether gaze location, assigned to either a face or non-face region of the display, varied between the Pixelated and Social movies. All models were fit using the Social condition as the reference event. Our baseline model found that probability of face-looking increased significantly with age (Δ-2LL = 96,983, Δdf = 3, p = 2.2 × 10^–1^), by about 50% each month. After adding a series of quality-control covariates to account for the quality of the data and eye-tracking recording (as detailed in “Methods”), we assessed the effects of stimulus condition on face-looking. Our best-fitting model indicated that, as expected, infants spent less time fixating on face regions during the pixelated condition (B = − 1.71, a 15.3% reduced probability of looking at faces, all else equal), compared to the same facial location during the social condition (Δ-2LL = 441,108, Δdf = 4, p = 2.2 × 10^–16^). Finally, a significant Age x Condition interaction indicated that this age-related change was seen primarily in the social condition (*Β* = − 0.004, Δ-2LL = 59, Δdf = 1, *p* = 2.2 × 10^–16^).

In addition to developmental changes in face-looking, we were interested in examining whether spontaneous changes in face-looking would correspond with changes in infants’ gaze complexity. To test this, we specified a second set of linear mixed effects models to examine whether, in addition to the effects of age and condition, there was a significant effect of the proportion of time spent fixating on faces on α. Only gaze data from the Social and Pixelated trials were included in these analyses, as there was no equivalent Area of Interest (AOI) in the Attention Cue trials.

**Figure 3:**
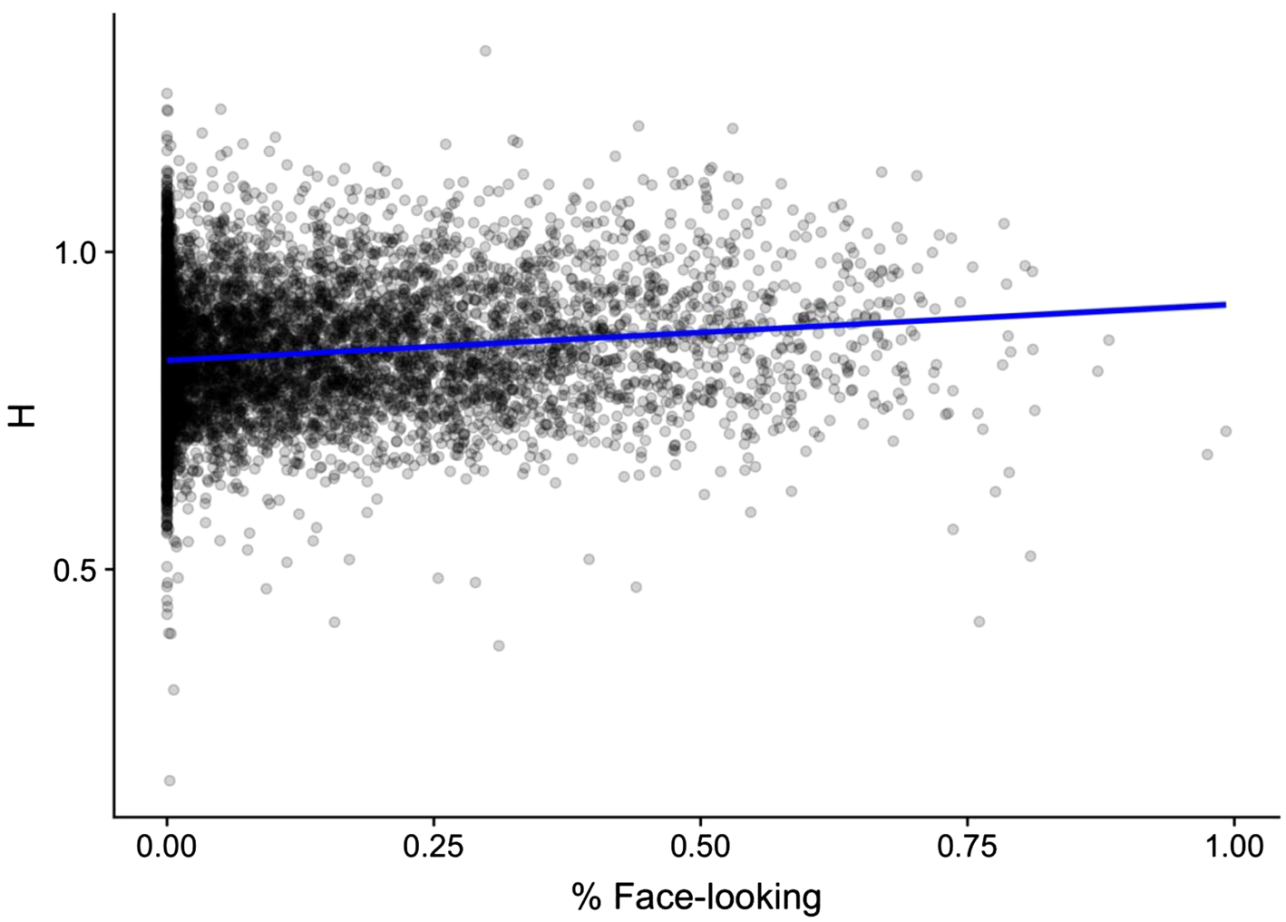
Fitted *α*-values plotted against Face-looking. Raw data are plotted, overlaid with best-fitting model results.

Our best fitting model indicated that on average, *α* values increased as a function of face-looking (Δ-2LL = 13.3, Δdf = 4, *p* = 0.00002) (see Table 2 for full model results). This effect was significant at all levels (i.e. average face-looking at the segment, movie, visit, and person level). This effect of within-visit face-looking on α did not vary as a function of age. Log-likelihood testing indicated that adding a Face-Looking × Condition interaction term did not significantly improve model fit relative to a model with Face-Looking alone (Δ-2LL = 2.7, Δdf = 4, *p* = 0.25).Model results overlaid on raw data can be found in Fig. 3.

**Table 2:** Linear mixed effects model table of model evidence displaying the effects of face-looking on α values with the Social condition as the reference event.

	Model 1	Model 2	Model 3	Model 4	Model 5
**Fixed effects**					
Between-person					
Intercept	0.834*** (0.006)	0.835*** (0.006)	0.839*** (0.005)	0.850*** (0.003)	0.848*** (0.003)
Avg Age			− 0.001* (0.001)	− 0.001* (0.001)	− 0.001* (0.001)
Face-looking%, person Avg					0.117*** (0.035)
Within-person, between-visits					
Age		0.003*** (0.000)	0.001** (0.000)	0.001** (0.000)	0.001** (0.000)
Face-looking (%), visit-Avg					0.087* (0.037)
Within-visit, between-movies					
Pixelated (dummy)				− 0.022*** (0.004)	− 0.017*** (0.004)
Face-looking (%), movie Avg					0.029* (0.012)
Within-movie, between-segments					
Face-looking (%), segment-Avg					− 0.027* (0.013)
**Random effects**					
Between-person					
Intercept	0.001	0.001	0.000	0.000	0.000
Age		0.000	0.000	0.000	0.000
Within-person, between-visits					
Intercept	0.002	0.002	0.001	0.001	0.001
Pixelated				0.001	0.001
Within-visit, between-movie					
Intercept	0.000	0.000	0.000	0.000	0.000
Residual	0.007	0.007	0.007	0.007	0.007
**Model fit indices**					
AIC	− 13,944.2	− 13,998.9	− 14,197.6	− 14,229.7	− 14,248.3
BIC	− 13,910.0	− 13,944.1	− 14,115.4	− 14,126.9	− 14,118.1
Log likelihood	6977.1	7007.5	7110.8	7129.9	7143.2

P < 0.001.

Coefficients are presented with standard errors in parentheses, with significant effects bolded for emphasis. Model 5, which includes a Face-looking effect is the final best fitting model.

## Discussion

The current study demonstrates that the developing visual attention system is dynamic and self-organized as early as 3 months of age. We observed these effects using DFA to elucidate the organization of infants’ gaze patterns across time and space. We show that infants’ gaze becomes more fractally self-organized over developmental time, when they are watching stimuli with social content, and when they spontaneously attend to faces.

Our findings support past empirical and theoretical work suggesting that fractal structure is a fundamental property of how healthy physiological and cognitive functions emerge from the interactions of many system components^42^. These results are consistent with a previous study documenting fractal structure of toddlers’ gaze patterns^17^ and provide important context to past work examining this property of gaze in adults. Adult gaze patterns exhibit fractal organization when viewing multiple types of stimuli^20,33^, and during complex visual search tasks^34^. Our finding that 91% of infant gaze patterns exhibited an organizational structure within the optimally flexible fractal range demonstrates that this system is already well organized in infancy, suggesting that the foundations of the visual attention system are already in place. These results complement extant findings of a striking level of maturity in higher-level visual cortex early in infancy^47^. Relatedly, other infant studies document patterns of visual foraging thought to support efficient information processing^48^, and that individual differences in visual attention are longitudinally associated with attentional and behavioral control in later childhood^49,50^.

Additionally, our finding that infants’ gaze becomes more self-organized over developmental time aligns with past work demonstrating that infants’ attention progresses from more dispersed and unpredictable to more focal and integrated over the first years of life^11^. This increase in organization could reflect a combination of more reflexive orienting as well as developmental advances in key visual attention networks, including increasing volitional control over behavior and the emergence of more adult-like visual perception. Our finding provides an important systems-view of the many developing components of early visual development that have thus far been studied quite separately (e.g.,^51^).

We also found that regardless of age, infants demonstrated more self-organized gaze patterns while watching the social movies, compared to other dynamic stimuli. We suggest that overall, the social movies may have elicited a more fractally organized gaze strategies from infants by virtue of their more engaging content and greater visual complexity.

Gaze patterns also exhibited increased fractal structure when infants spontaneously looked to faces. This finding was true both between-infants (e.g., infants who looked at faces more than the group average had increased fractal organization) and within-infants (e.g., infants who showed increased face-looking during segments, movies, and visits relative to their own averages had increased fractal organization during this segments, movies, and visits). Contrary to our hypotheses, this effect held in both the Social and Pixelated movies, perhaps because while the social visual information is degraded in the Pixelated condition, it can still be inferred.

Our findings suggest that not only does the fractal organization of gaze change between conditions, but this measure also varies dynamically on a shorter timescale as a function of infants’ gaze to highly relevant features. These findings highlight how fractal organization emerges at the intersection between infants’ attentional abilities and the nature of the stimuli themselves^14^. Evidence that the fractal structure of infants’ gaze varies by the salience of the overall stimulus and spontaneous looking to faces suggests that the fractal organization of gaze may reflect a state of active engagement with salient stimulus features.

This interpretation is consistent with findings that fractal structure may index attentional and cognitive effort in adults (e.g.,^36^), as well as with findings that the fractal structure of relevant adult systems is associated with metrics of on-task performance within a variety of different tasks and systems, using a host of different methods. For example, the fractality of adults’ eye gaze is associated with performance on complex visual search^43^ and language comprehension^52^ tasks. A study examining coordinated movement found that the fractal structure of timing errors varied by the employment of different effortful cognitive strategies during the task^53^. One small study found increased fractal structure of blood oxygen level dependent (BOLD) activity when participants recalled emotional relative to neutral memories^54^. Such a metric of psychobiological system engagement in studies of infant development would have utility in its ability to quantify aspects of experience, such as perceived salience, that have often been elusive in the context of scientific inquiry. Of course, further evidence is needed to support this interpretation of fractal structures in infants’ eye-gaze. To provide convergent evidence of fractal structure indexing attentional and cognitive efforts in infants, future work will measure multiple systems implicated in visual attention (e.g., autonomic nervous system and neural activity) and will employ tasks that directly measure attention performance. Further, a developmental psychopathology approach comparing typically developing children and those at risk for neurodevelopmental disorder may further elucidate the importance of fractal structures for human psychobiology and shed light on their potential clinical utility as early biomarkers.

Our findings should be considered in light of several limitations. One limitation of our stringent quality-maximizing exclusionary process is that our final sample resulted in an average of only two time-points per participant, limiting our ability to model age-related change in complexity within a person. However, 52% of our final sample did contribute two time-points or more. Second, for our hypothesis about the fractal structure of gaze patterns while viewing different stimulus contexts, we were unable to control for all characteristics of the stimulus conditions. While the Social and Pixelated stimulus conditions were matched on some lower level properties (e.g., motion), they may differ along other dimensions such as contrast and color. It could be the case that lower-level properties of the stimuli could account for some of our between-condition findings.

Finally, we recognize both the benefits and costs to our analytic approach. On one hand, measuring fractal organization allows one to eschew reductionism and capitalize on potentially meaningful variability inherent to emergent phenomena (e.g.,^26,43,55^). Emergent properties are thought to reflect multiple interacting components that change each other’s dynamics in their interactions^26^, thus amount to more than the simple sum of component parts. This is particularly noteworthy, given the dynamic systems thinking at the heart of many contemporary developmental models. DFA is also agnostic to the specific nature of the system under study and can be applied to any level of analysis, from cellular to behavioral dynamics. Additionally, this approach also allows researchers to account for organization occurring at multiple timescales, without making a priori assumptions about the temporal nature of relevant component processes.

On the other hand, this approach is not without criticism. Systems-level analyses are a bit of a ‘black box’; the specific processes involved remain rather opaque both within, as well as between, individuals. That is, while these metrics quantify system organization, they tell us nothing about which systems this organization reflects, the potentially differential contributions of oculomotor mechanics (e.g., micro saccades), nor whether two identical fractal metrics between individuals executing the same task involve similar constituent parts. Thus, this approach is meant as a complement to, not a replacement of, other approaches designed to test subcomponents of a system in order to understand changes in behavior. Furthermore, like any other summary statistic, indices of system complexity are likely imperfect summations of the inherent dynamics of interest. Despite these challenges, we believe that this approach proves useful for helping to answer a variety of scientific questions about the emerging organizational structure of psychobiological systems thought to support complex behavior, while recognizing the open questions that remain. It catalyzes important future lines of inquiry including questions about the ways in which fractal organization of gaze may shape functional behavioral outcomes and interact with other systems.

In conclusion, this study employs a novel dynamic systems approach to quantify the fractal organization of children’s moment-to-moment gaze patterns as they viewed a variety of stimuli. Our findings suggest that the visual attention system is well organized within the first 3 years of life. Complex, fractal organization is evident even with very young infants—particularly in the context of socially salient stimuli—and these organizational properties show continued growth throughout toddlerhood. In addition to informing our understanding of normative developmental processes during this span, the present findings have the potential to clarify the early emergence of atypical social-attentional biases common to a number of neurodevelopmental disorders (e.g., autism spectrum disorder).

## Methods

### Original sample

14,870 time-series of eye-tracking data were collected from 190 1.84- to 35.04-month-old infants (86 females, mean age = 10.62 months) across 421 visits to the lab. All participants were recruited from the Institute of Child Development’s participant registry at the University of Minnesota as a part of a larger mixed cross-sectional and longitudinal study. Participant exclusion criteria included: (1) history of known genetic syndromes associated with ASD risk; (2) significant medical conditions affecting growth, cognitive development, or significant vision or hearing impairment; (3) birth weight < 2000 g and/or gestational age < 36 weeks; (4) history of significant perinatal adversity, or exposure in-utero to neurotoxins; (5) contraindication for magnetic resonance imaging; (6) having been adopted; and (7) family history of a first-degree relative with intellectual disability, autism, psychosis, schizophrenia, or bipolar disorder. Parents provided written and informed consent for their child’s participation in the study. All protocols are in accordance with relevant guidelines and regulations and were approved by the University of Minnesota’s Institutional Review Board (IRB).

As part of a planned missingness design^56^, children contributed between 1 and 6 waves of data across this age span (mean 2.22 waves) during visits to the lab. Our final sample for analyses was comprised of children from 3 cohorts, A, B, and C, as shown in Figure S1 of the Supplementary Information. Cohorts A and B were recruited into accelerated longitudinal studies. Cohort A consisted of 70 infants with a mean age of 14.10 months (enrollment age range of 1–35 months), with study length commitments of 1 visit to 3 years. Cohort B consisted of 90 infants with a mean age of 8.98 months (enrollment age range of 3–6 months), with a study length commitment of 2 years and five behavioral visits. Cohort C consisted of 23 infants with a mean age of 9.53 months, recruited for cross-sectional visits between 6 and 15 months of age. Cohort dummy variables were tested in the linear mixed effects models but did not predict any variance in our dependent variables (*p*’s > 0.5).

### Experimental set-up

**Figure 4:**
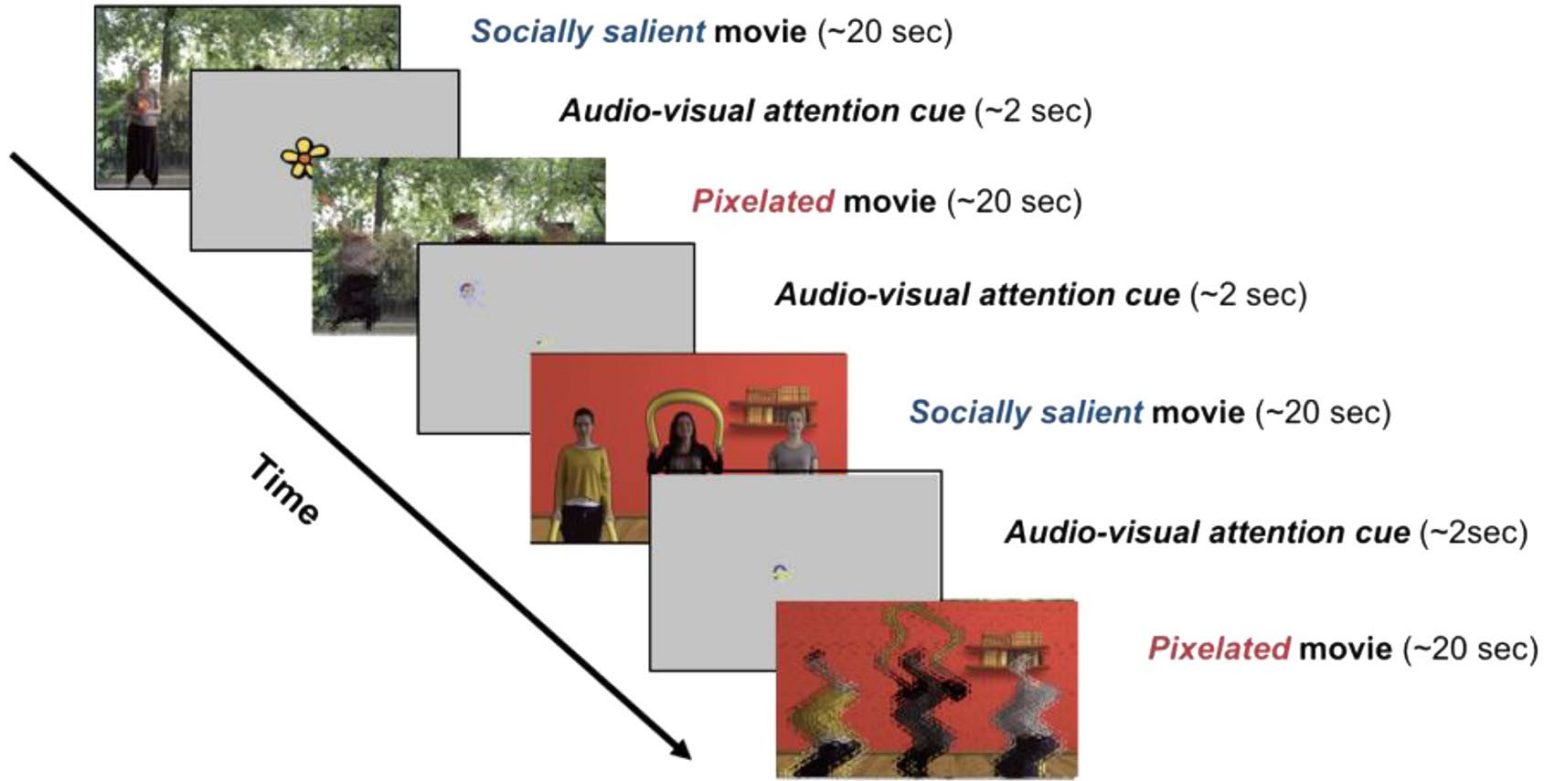
Example of the sequence of stimuli presented at each visit. Socially salient movies featured women moving with child-friendly objects and the pixelated movies featured blurred, or pixelated, versions of the women moving with child-friendly objects.

At each visit, infants were seated in their parent’s lap approximately 65 cm from a 27-in. 1920 × 1080 resolution ASUS monitor that subtended 43.6° of visual angle with an aspect ratio of 16:9. They watched four 20-s movies of women dancing to lively music while waving toys, as well as pixelated versions of these same videos (from^57^). These two stimulus conditions (Social and Pixelated; Fig. 4) were used to compare the fractal structure of gaze patterns while viewing social stimuli, relative to stimuli with most of the social information degraded. Movies were interleaved with dynamic audio-visual attention cues (Fig. 4) used for estimating recording accuracy and precision, and for establishing baseline levels of infants’ gaze organization^58^.

(A) *Social movies*. Movies of naturalistic social stimuli, featuring 3 women dancing at intermittent intervals (Fig. 4); (B) *Pixelated movies*. Pixelated versions of the Social movies were used to blur the social information, thereby reducing the social salience of the scene while preserving some lower-level visual and motion properties (Fig. 4); (C) *Audio-visual attention cues*. Animated target stimuli were presented on a gray background (R: 192 × G: 192 × B: 192, Hue: 160 Lum: 181), accompanied by attention-capturing sounds (Fig. 4). Cues were made up of a green circle (diameter 0.63° at a viewing distance of 60 cm), surrounded by 3 annuli that increased in size by 0.63°. The largest annulus (blue) was 2.52° in diameter. The stimuli were placed in the four corners of the screen at a distance of 14.1°, 8.2° (480, 270 pixels) from the edge of the screen, at a resolution of 1920 × 1080 pixels. The fifth target was located center-screen (960, 540px).

The Social and Pixelated movies were accompanied by Basque children’s music, a language that none of our sample had exposure to. There were 4 different Social movies, each with a Pixelated counterpart, for a total of 8 movies interspersed with audio-visual attention cues, as shown in Fig. 4. As shown in Table 1, there was very little variance at the movie level, suggesting that alpha values do not vary significantly by movie. At each visit, infants were randomly assigned to 1 of 2 pseudo-random movie presentation orders, in which either a Social or Pixelated movie was presented first (version order type did not predict any variance in our outcomes (p’s > 0.5)). The entire eye-tracking task lasted approximately 5 min.

### Eye-tracking data collection

At each visit, infants’ eye movements were recorded with non-invasive corneal-reflection binocular eye-tracking equipment (Tobii TX300, recordings sampled at 300 Hz; Tobii Studio; Tobii Technology, Danderyd, Sweden). Because it is recommended that DFA use at least 1000 samples of contiguous data^19^ and could be susceptible to noise artifacts, the processing of eye-tracking data was done with two goals in mind: to be stringent about eye-tracking data quality, and to create time-series with enough contiguous raw data to analyze.

### Quality-control exclusion criteria

Infants’ eyes were calibrated to the eye-tracking equipment at the beginning of each visit, using the manufacturer’s five-point calibration procedure. Precision, or the distance between repeated samples of gaze points^58^, was included given that it is a measure of variability and thus is likely to influence DFA calculations that quantify nested patterns of variability. To measure the eye-tracker’s precision throughout the experiment, experimental trials were interleaved with audio-visual attention cues. Precision was included as a quality-control exclusion criterion due to its potential to influence fractal structure, given that it is an index of variation around a target stimulus. Audio-visual attention cues were presented at 3 time-points during each eye-tracking visit: at the beginning, interleaved with the Social and Pixelated movies, and at the end. Infants’ longest contiguous raw eye-tracking data for all available Attention Cue trials were analyzed for precision. Data from eye-tracking sessions with Root Mean Square Error of Approximation (RMSEA) values two standard deviations or more above the sample mean (indicating poor precision) were excluded. As such, data from eye-tracking sessions with an average RMSEA (averaged across X and Y axes for both eyes) greater than 1.21 degrees of visual angle were excluded from analyses. As a result, data from 5 entire participants, 28 entire eye-tracking visits, totaling 939 time-series were excluded from future analyses. For the remaining eye-tracking visits, the average precision was 0.43° of visual angle.

### Time-series generation

**Figure 5:**
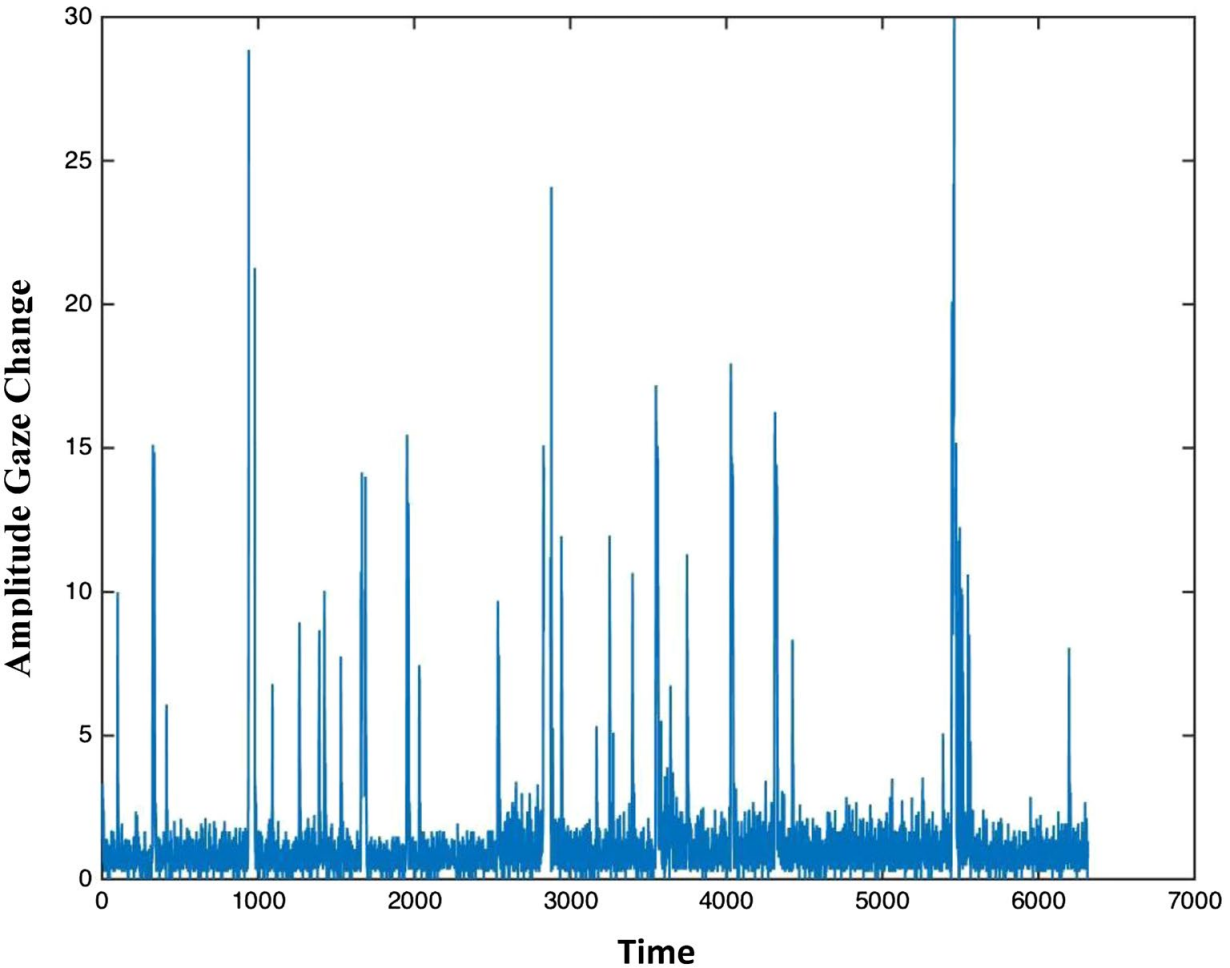
Example of a time-series comprised of the amplitude of X- and Y-coordinate gaze change (calculated as the change in Euclidian distance between two samples ${\text{D}} = {\text{sqrt}}\left( {\left( {{\text{X}}_{2} - {\text{X}}_{1} } \right)^{2} + \left( {{\text{Y}}_{2} - {\text{Y}}_{1} } \right)^{2} } \right)$, relative to the change in time between samples, $T={t}_{2}-{t}_{1}$ for the entire data stream, $D/T$) over time (1/300th of a second from a 300 Hz sampling rate).

After the eye-tracking data were collected, we created gaze-based time-series using the amplitude of change in infants’ raw gaze position, sampled every 3.33 ms, over time (as shown in Fig. 5). Raw, unfiltered eye-tracking data were used given: (A) the dynamic systems theoretical motivations for this study, (B) our aim to examine the temporal unfolding of gaze organization at multiple different timescales, and (C) the input requirements for DFA analyses. Time-series were generated for infants’ eye movements while watching the Social and Pixelated stimulus conditions as well as while watching the audio-visual attention cues to serve as a baseline measure of gaze organization for an exogenously driven spatial attention cue.

As implemented, DFA does not allow for missing data points. To maximize the number of usable time-series, eye-tracking data from each movie were divided into approximately 6-s segments for analysis. Each Social movie was viewed frame-by-frame in Datavyu (Datavyu, Version 1.0) to segment the movie into discrete, 3- to 8-s movement events (e.g., one woman begins dancing and the others sequentially join her; all women sequentially stop moving; one woman dances and then stops and then starts again; one woman moves her balloon and then starts and stops dancing). Of the 4 movies, 3 were divided into 3 segments (mean 7.38/SD = 2.15 s long), and 1 movie was divided into 4 segments (mean 6.47/SD = 1.58 s long) based on events in the movies. All Social movies and their Pixelated counterparts were segmented for analysis, as was each audio-visual attention cue.

Blinks were identified using a noise-based algorithm^59^. All data missing as a result of blinks (less than 200 ms) were linearly interpolated using data from the last valid sample before the start of the blink, and the first valid sample after the end of the blink (mean proportion interpolated = 0.073, range 0–0.76). After interpolation, infants had an average proportion of 0.15 (range 0–1) residual missing data per segment. Given past work suggesting too much aggregation can bias detrended fluctuation analysis (e.g.,^20^), we chose a stringent quality control threshold for the permissible proportion interpolated for each segment. After visualizing the distribution, the 80th quantile threshold for the sample’s proportion interpolated was calculated (0.115) and segments with a proportion exceeding that value were excluded from analyses; data from an additional 1 entire infant, 1 entire eye-tracking session, totaling 2788 time-series were removed from future analyses. The mean proportion of interpolated segments for the remaining sample was 0.0152. Proportions of interpolated and residual missing data were entered into all models as covariates.

Lastly, we identified each participant’s longest contiguous stream of eye-tracking data for each movie segment, and calculated the change in Euclidian distance between two samples ${\text{D}} = {\text{sqrt}}\left( {\left( {{\text{X}}_{2} - {\text{X}}_{1} } \right)^{2} + \left( {{\text{Y}}_{2} - {\text{Y}}_{1} } \right)^{2} } \right)$, relative to the change in time between samples, $T={t}_{2}-{t}_{1}$ for the entire stream. The amplitude of gaze change was then calculated as $D/T$ as shown in Fig. 5 and used for our time-series. We chose to use the amplitude of change in gaze position over time as our time-series in order to account for changes on both the X and Y axes, to avoid excessive computation and difficulties interpreting our outcome for fractal organization along just one axis (in alignment with^15,34,35^). As DFA has not been validated on time-series with fewer than 1000 data points^19^, we excluded all time-series with fewer data points. Accordingly, data from an additional 2 participants, 8 eye- tracking visits, totaling to 2223 segments were excluded from future analyses.

### Final sample for analysis

The final sample consisted of 182 infants (84 females) who completed a total of 384 eye-tracking visits, amounting to 8920 time-series. The final sample had an average age of 15.33 months (range 2.50–35.04 months) and contributed data at an average of 2.04 visits (range 1–6). The final sample of infants watched an average of 10.29 movies per visit (range 1–16; including audio-visual attention cues), contributing an average of 22.23 time-series of data per visit (range 1–36). On average, infants contributed 9.16 (range 1–14) time-series from Pixelated movies, 10.43 (range 1–14) time-series from Social movies, and 5.14 (range 1–8) time-series from audio-visual attention cues, at each visit. The average length of each time-series was 5775.237 data points long (1925.712 ms; SD = 2013.26). Neither the average numbers of Pixelated or Social time-series explained any variance in our outcomes. The final sample had an estimated average eye-tracking accuracy of 1.76 (range 0.29–5.88) and precision of 0.40 (range 0.077–1.19) in degrees of visual angle**.** A flow diagram of the exclusionary process is shown in Figure S1 of the Supplementary Information.

### Detrended fluctuation analysis (DFA)

A variety of algorithms are available to estimate parameters that coincide with this noise distribution, each often a linear or non-linear transform of the other^60^. We chose DFA as it has been established for biomedical data and, unlike other metrics of entropy or variability, this approach allows us to examine variability at multiple time scales. This property of scale invariance is thought to reflect interaction-dominant characteristics and potentially self-organized systems that are core to developmental dynamic systems theories long heralded by the field but usually left unmeasured. DFA was performed on the time-series (amplitude of gaze change over time) derived from each movie segment, using a MATLAB package created specifically for biomedical time-series^19^. This analysis estimates the power law exponent that defines the scale-invariant, or fractal, structure of a time-series through the following analytic sequence. The user-defined parameters, values, and respective justifications are listed in Table S1 of the Supplementary Information.

First, the time-series (amplitude of X and Y coordinate gaze change over time) is converted to a random-walk-like structure by subtracting the mean value and then taking the integral. Next, the time-series is divided into 4 equal-sized non-overlapping windows, with a minimum window size of 4 and a maximum window size of ¼ of the length of the time-series, and a polynomial trend is fit to each window of data. The local root mean square (RMS) is then computed for the residual variation around a specified polynomial trend (m = 2) fitted to each window of data. Given that fast- and slow-changing fluctuations in a time-series influence the RMS differentially depending on the window size (i.e., fast-changing fluctuations influence small window sizes and slow-changing fluctuations influence larger windows), the RMS is then calculated for the different window sizes.

**Figure 6:**
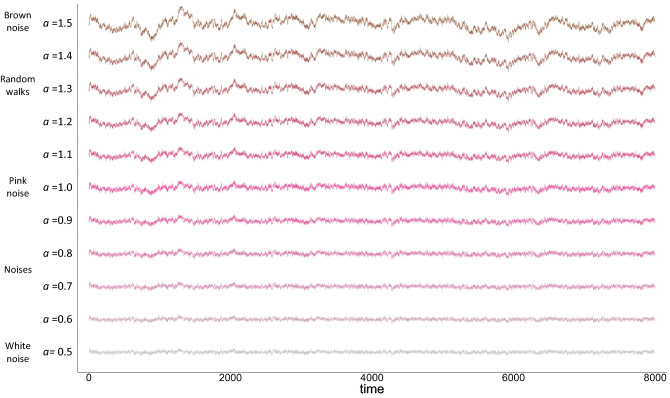
Visualization of time-series with different organizational structures from white noise, through pink noise, to brown noise with corresponding *a* values*.*

DFA identifies the monofractal structure of the time-series as the power law relation between the overall RMS’s computed for multiple window sizes (as shown in Figure S3 of the Supplementary Information). This power law relation is indexed by *a* or the slope of the regression line fit to the log(frequency) and log(power) of the variation in the time-series. *a* denotes how fast the local RMS changes with increasing window sizes, summarizing the long-term memory of the series and quantifies the monofractal structure of a time-series on a continuum from white noise (*a* ~ 0.5), through ‘pink noise’ (*a* ~ 0.8), to brown noise (*a* ~ 1.5) as shown in Fig. 6. *a* values calculated using a larger number of windows (i.e., 19 as in^19^) are comparable with r = 0.8 and a median absolute difference of 0.04 (range 0–0.6).

### Linear mixed effects modeling

**Figure 7:**
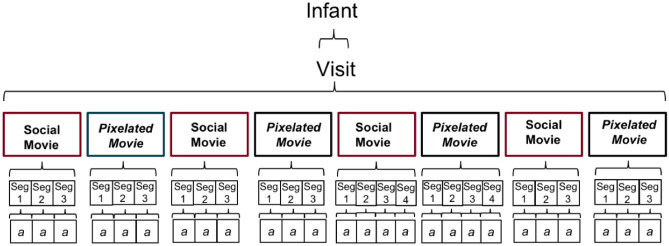
Illustration of the nested levels of the data. Infants came into the lab for multiple visits at which they viewed two kinds of movies. Each movie was then divided into segments for fractal analyses that were used to create time-series for DFA analyses. *a* values were derived for each time-series of data.

We conducted linear mixed effects modeling using the lme4 package in R 3.3.1, to account for the fact that our dependent variable, *a*, is nested within participants, eye-tracking visits, and movies (as shown in Fig. 7). Given that time-series (level 1) were nested within movies (level 2), which were nested within laboratory visits (level 3), which were nested within individual participants (level 4), we tested a 4-level nested random effects structure for all of our models.

The following quality control exclusion criteria covariates that provide indices of the quality of the eye-tracking data were tested at all appropriate levels: the proportion of interpolated data, the length of the longest contiguous sequence of gaze data used for DFA, and the estimated precision of the gaze recording. The following between-person covariates were tested: sex, cohort, average age of data contribution (centered at the grand mean), average eye-tracking precision (centered at the grand mean), average proportion of data interpolated (centered at the grand mean), and average length of the longest fixation used for DFA calculations (centered at the grand mean). The following between-visit covariates were tested: estimated eye-tracker precision (centered at the person mean), average proportion of the data interpolated (centered at the person mean), average longest fixation used for DFA calculations (centered at the person mean), the number of Pixelated and Social movies watched, and counterbalancing-version (denoting the order of movies presented). The following between-movie covariates were tested: average proportion of the data interpolated (centered at the visit mean), and the average longest fixation used for DFA calculations (centered at the visit mean). Finally, the following between-segment covariates were tested: proportion of the data interpolated (centered at the movie mean), and the longest fixation used for DFA calculations (centered at the movie mean).

Linear mixed effects models were fit in the following steps: after establishing the appropriate functional form (i.e., how *a* values change with age), the appropriate structure of random effects was assessed (i.e., whether random effects for both intercept and slope were needed). Given their ages and the nature of the task, we did not expect that even the oldest children would move into the brown-noise range but we nonetheless tested non-linear age effects, as a sensitivity check. Person-level random effects for intercept and slope, visit-level random effects for intercept and stimulus type, and movie-level random effects of intercept were included in the model. Next, quality-control covariates (e.g. eye-tracker precision) were added to the model. Finally, categorical variables for stimulus types (i.e., Social, Pixelated, and Attention Cue), and their interaction with age, were added to test our hypotheses. The Social movie dummy variable was omitted from the model and was thus used as the reference event. At each step, model comparisons were conducted using chi square log likelihood ratio tests and Second-Order Akaike Information Criteria (AIC; accounting for sample size and model complexity). Additional model parameters were retained only if AIC values and likelihood ratio tests indicated that adding them led to a model that better fit the data. Percentages of variance explained were calculated by squaring the correlation of fitted and actual values.

To test the association between *a* and spontaneous face-looking, only data from Pixelated and Social movies were included in the linear mixed effects models (n = 7019 eye-tracking segments). The Social movie condition was used as the reference event and omitted from the model. Linear mixed effects models were fit using the same steps as above, with the addition of four variables allowing us to examine effects of face looking at all four levels: between-person face-looking (centered on the grand mean), within-person face-looking (visit average, centered on the individual’s average face-looking), within-visit face-looking (movie average, centered on the visit’s average face-looking), and within-movie face-looking (segment average, centered on the movie’s average face-looking). We also tested for interactions between Face-looking and stimulus Condition, as we hypothesized that face-looking should be related to α only in the Social movie condition.

### Defining areas-of-interest (AOI’s)

Open Source Computer Vision Library^61^ was used to automatically identify face-related Areas-of-Interest (AOIs) for each movie-frame in the non-pixelated movies. After face regions were detected in these movies, we hand-checked each movie frame to ensure that each face was accurately detected. If there were errors (e.g., partially obscured faces were often missed by the algorithm), we linearly interpolated the correct face AOI from the most recent frames before/after the missing frame. If the face was completely obscured by an object (e.g. a ball), that face AOI was not included for that frame. Thus, the number of face AOI’s in each frame ranged from 0 to 3. AOI’s from each Social movie were then copied to their Pixelated counterpart.

### Estimating age-related change in face-looking

Eye-tracking data from time-series that contributed to DFA were also examined for the number of samples in face AOI’s versus non-face AOIs. For each time-series, we tallied the number of gaze coordinates recorded within the boundary of a face-AOI, and the number of gaze coordinates recorded outside the boundary of a face-AOI. Then, we calculated the proportion of time spent within a face AOI relative to anywhere else (i.e., $[\mathrm{n\ samples\ in\ face\ AOI}] / [\mathrm{n\ samples\ in\ nonface\ AOI}])$ across all movie segments in a visit.

We used generalized mixed effects logistic regression models to estimate age-related change in infants’ spontaneous preferences for faces in a given movie. The outcome variable for these analyses was the number of samples in a face AOI versus the number of samples in a non-face AOI, and a logistic linking function was used. As with our other models, we first established the functional form, then added covariates to the model, and then examined the effect of stimulus condition (Pixelated or Social) and interactions with age. Random effects were included at the person-level only, as models including them at the visit-level failed to converge. The Social condition was used as the reference group.

The best fitting-model included random effects of intercept, age, and stimulus condition, and fixed effects of intercept, age, stimulus condition, and Age x Condition. Eye-tracker precision, the duration of the longest stream of contiguous data, and the proportion of interpolated data for the time-series were included as significant quality control covariates, in addition to the individual’s average age across visits to account for the accelerated longitudinal design.

## Supplementary information

Supplementary Information 1

## Data Availability

Data from this study are available upon request. Code is available at https://github.com/rrobinn/fractal-eye-analyses/.
