# Supplementary material for: Infants’ gaze exhibits a fractal structure that varies by age and stimulus salience
Source: Sci Rep. 2020 Oct 14;10:17216. doi: 10.1038/s41598-020-73187-w (PMC7560596; doi:10.1038/s41598-020-73187-w)
Supplement: Supplementary file 1 — Supplementary Information 1 [file 41598_2020_73187_MOESM1_ESM.pdf]

Supplementary Information

Title

Infants' gaze exhibits a fractal structure that varies by age and stimulus salience

Authors

Isabella Stallworthy[1†], Robin Sifre[1†], Daniel Berry[1†], Carolyn Lasch[1], Tim Smith[2], & Jed Elison[1,3]

[†]*shared first authors*

Author Affiliations

1. Institute of Child Development, University of Minnesota, USA
2. Department of Psychological Sciences, Birkbeck University of London, UK
3. Department of Pediatrics, University of Minnesota, USA

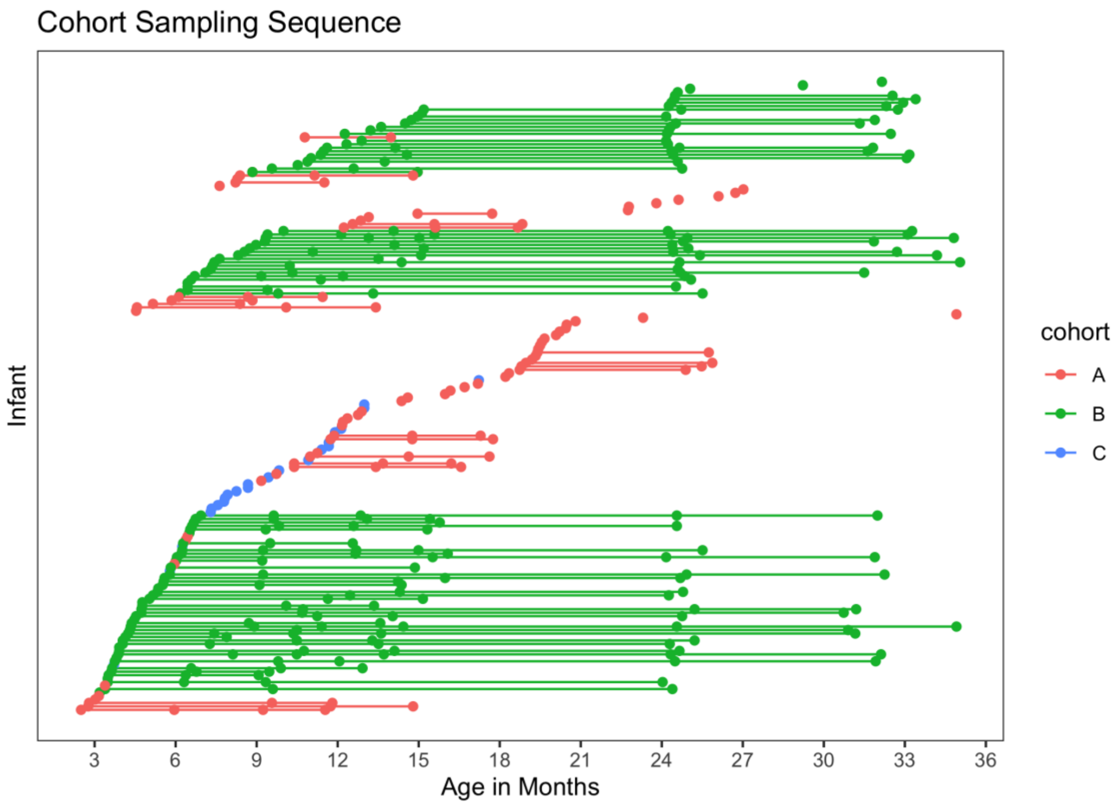

Figure S1. Data collection points for each infant over developmental time for each of the three cohorts of the accelerated longitudinal design.

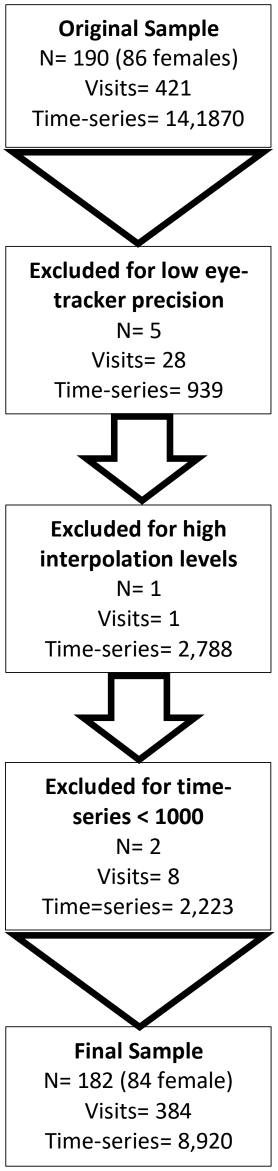

Figure S2. Flow diagram depicting characteristics of the original sample and the participants sequentially excluded for low eye-tracker precision, high levels of interpolated data, and time-series <1,000.

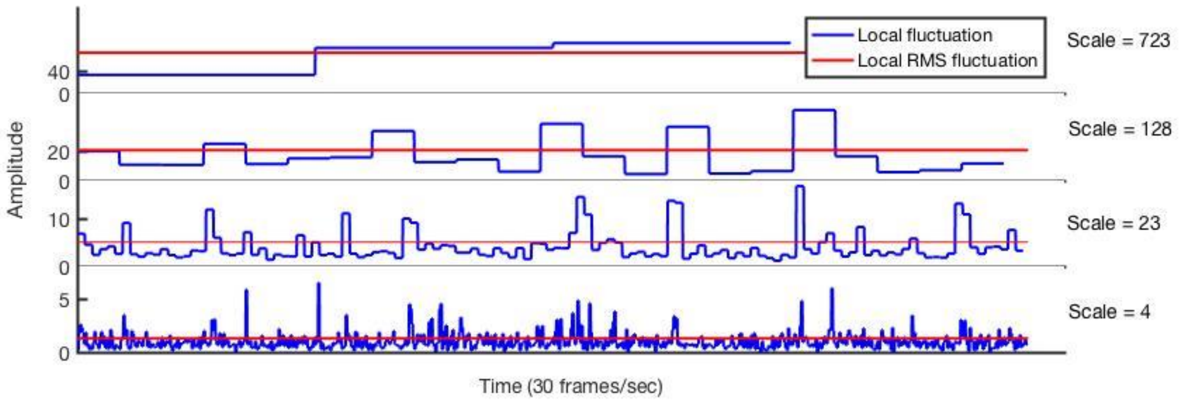

Figure S3. Example illustration of the local fluctuations in gaze patterns at different measurement scales as part of the DFA processing steps.

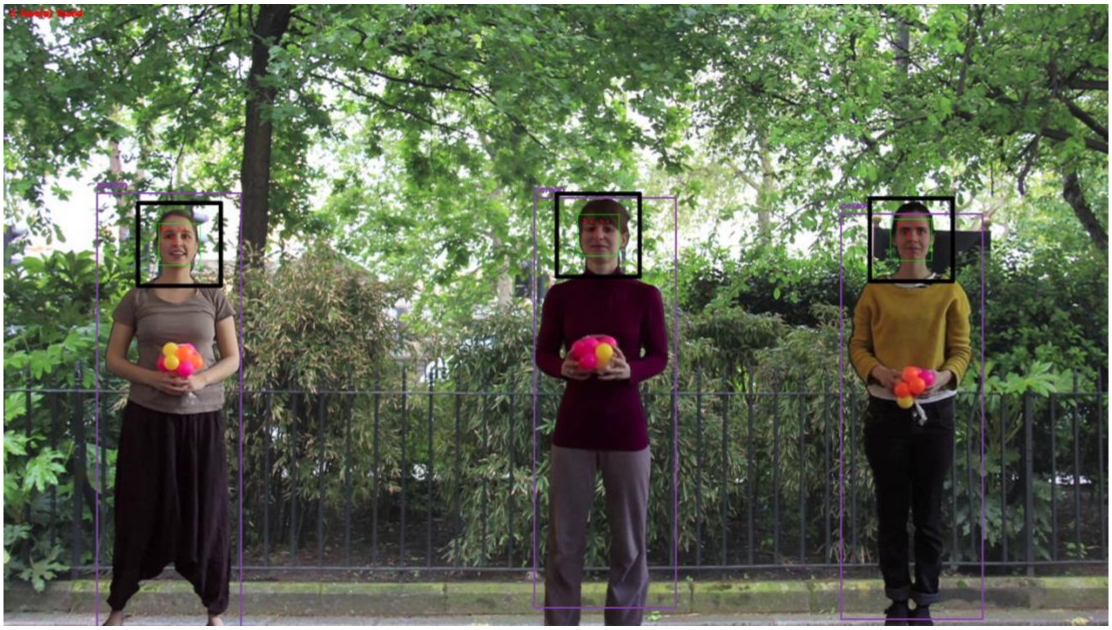

Figure S4. Bounding boxes of faces generated by OpenCV (in green) were expanded to create the face AOI's (in black) and used for examining within-person changes in face-looking and visual complexity.

Table S1. User-Defined DFA Parameters for all Fractal Analyses.

Parameter	Description	Value	Source/Justification
m	Polynomial order for detrending at each window	2 (quadratic)	Linear log2(scale)v log2(Fq)) plot indicates scale invariance compared to other m values
scmin	Minimum window size for detrending	4	Wallot et al., 2015; Coey et al., 2012
scmax	Maximum window size for detrending	(Length of the time-series)/4	Ihlen, 2012
scres	Total number of window sizes with which to detrend and calculate local RMS	4	Accommodates time-series closer to 1000 data points long.
